# A study on the therapeutic effects of biplane skin dilator implantation in auricular reconstruction

**DOI:** 10.1038/s41598-021-00179-9

**Published:** 2021-10-15

**Authors:** Pengfei Sun, Meng Lu, Changchen Wang, Bo Pan

**Affiliations:** Department of Plastic Surgery, Plastic Surgery Hospital, Chinese Academy of Medical Sciences and Peking Union Medical College, No.33 Badachu Road, Shijingshan District, Beijing, 100144 China

**Keywords:** Medical research, Outcomes research, Clinical trial design

## Abstract

This study aimed to compare the therapeutic effects of biplane skin dilator implantation with those of conventional skin dilator implantation in auricular reconstruction. A total of 137 patients with microtia who met the inclusion criteria from January 2020 to April 2021 were retrospectively selected. Sixty-three patients comprised the control group and were implanted with a skin expander using the conventional method. Seventy-four patients comprised the experimental group and were implanted with a skin expander using the biplane method. Non-parametric tests were used to compare the down-moving distance of the skin dilator between the experimental group and the control group. There was a statistically significant difference in the down-moving distance of the skin dilator between the experimental group and the control group (*P* < 0.05). The chi-square test showed no significant difference in postoperative complications between the experimental group and the control group (*P* > 0.05). Moreover, there was no significant difference in the satisfaction rate of patients and their families between the experimental group and the control group (*P* > 0.05). In this study, the treatment effect of biplane skin dilator implantation was better than that of conventional skin dilator implantation.

## Introduction

Microtia is a common organ malformation^1^. At present, the main treatment for microtia is ear reconstruction^2^. Skin dilators play an important role in ear reconstruction^3^. In the 1950s, Neumann, who was an American plastic surgeon, first used skin dilators for auricular reconstruction. Chinese plastic surgeons began to use skin dilators in 1984. In 1993, Professor Ai Yufeng reported the clinical experience of Chinese patients undergoing auricle reconstruction using skin dilators^4^. In 2006, Professor Zhuang Hongxing summarized the treatment experience of 3000 patients who underwent auricular reconstruction using skin dilators, and divided auricular reconstruction into three stages^5^. In the first stage, a skin dilator was placed into the subdermal layer of the mastoid region behind the ear. In the second stage, a dilated flap and a retroauricular fascial flap were used to cover the scaffold carved from the costal cartilage of the patient. In the third stage, the subunits of the reconstructed ear were repaired to perfect the morphology of the reconstructed ear. At present, this three-stage ear reconstruction method is the main surgical method for auricular reconstruction in China^6^.

However, in the course of using this third-stage ear reconstruction method, we found that with an increase in sterile saline injection, the gravity of the skin dilator and the tension of the skin above the skin dilator cause the skin dilator to move downward. The result of this downward movement is insufficient expansion of the skin above the skin dilator and greater expansion of the skin below the skin dilator. This problem increases the difficulty of auricular reconstruction and affects the appearance of auricular reconstruction. To solve this problem, we used the biplane method to implant the skin dilator in the first-stage operation. We conducted this study to compare the effect of biplanar skin expander implantation and conventional skin expander implantation on skin expansion in the mastoid region behind the ear.

## Methods

This study was reported in accordance with the Strengthening the Reporting of Observational studies in Epidemiology (STROBE) statement^7^.

### Clinical data

The clinical and postoperative data of patients with microtia who underwent skin dilator implantation at the Plastic Surgery Hospital from January 2020 to April 2021 were collected. In this study, 137 patients with microtia met the inclusion criteria. Patients who underwent the biplanar method during the first stage of surgery were included in the experimental group (n = 63), and patients who underwent the conventional method during the first stage of surgery were included in the control group (n = 74).

In the experimental group, there were 38 male patients and 25 female patients, with an average age of 10.08 ± 2.64 years. The experimental group included 41 cases of microtia on the left and 22 cases of microtia on the right. In the control group, there were 43 male patients and 31 female patients, with an average age of 10.16 ± 2.52 years. The experimental group included 38 cases of microtia on the left and 36 cases of microtia on the right. Patients in the experimental group were followed up 61.81 ± 5.55 days after the first-stage operation, and patients in the control group were followed up 62.01 ± 6.09 days after the first-stage operation. There were no significant differences in sex, age, location of disease, and follow-up time between the two groups (*P* > 0.05).

### Ethics approval and consent to participate

Patients and their families provided written informed consent for participation in the study. The patients/legal guardians agreed to use their images for publication of this article and signed the informed consent. All procedures performed in studies involving human participants were in accordance with the ethical standards of the institutional and/or national research committee and with the 1964 Helsinki Declaration and its later amendments or comparable ethical standards. This study was approved by the Medical Ethics Committee of Plastic Surgery Hospital, Chinese Academy of Medical Sciences (file no. 2020-186).

### Inclusion and exclusion criteria

The inclusion criteria were as follows: unilateral microtia, good health, no other chronic diseases except auricular deformity, a normal mastoid area anatomy without skull depression.

The exclusion criteria were as follows: bilateral microtia; serious heart, kidney, liver, or other organ diseases; severe hemifacial microsomia; an abnormal mastoid area, such as an abnormal skull structure or an abnormal skin color.

### Surgical methods

**Figure 1:**
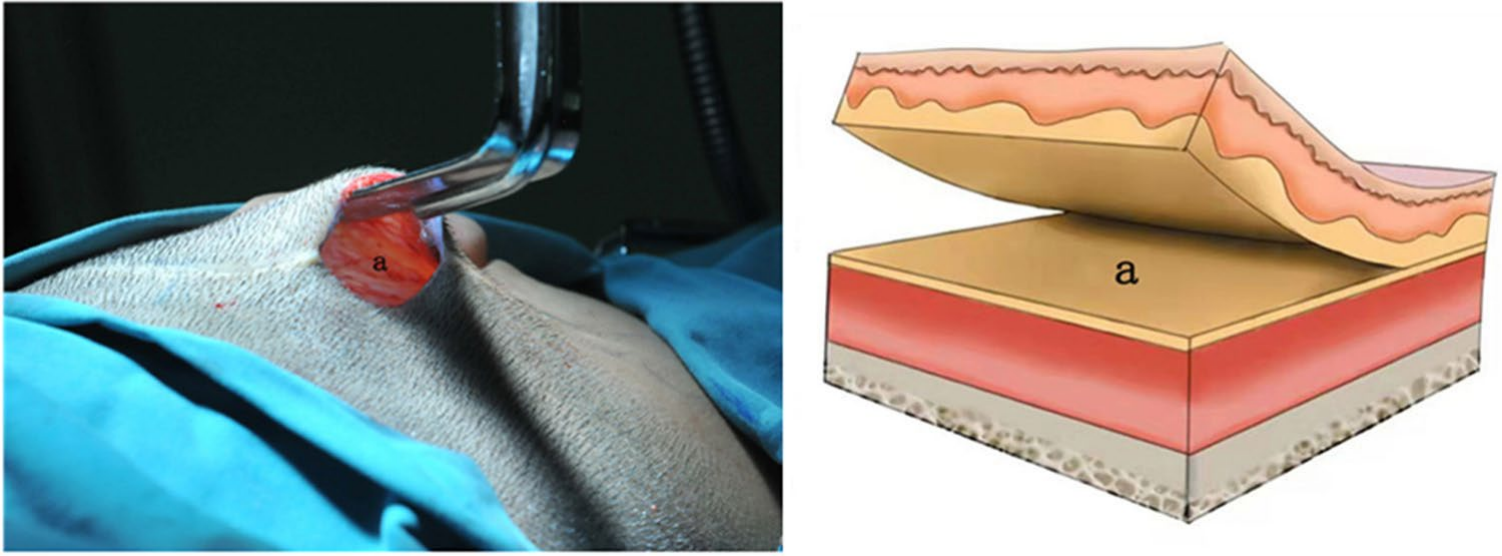
Schematic diagram of the dissection layers in the conventional surgical procedure. a: The superficial fascial surface.

In the control group, patients were treated with a skin dilator by conventional surgery. The incision was located 0.5–1 cm behind the patient’s hairline and was approximately 3–5 cm long. The surgical area was designed according to the bare skin area without hair growth in the mastoid region. After local anesthesia with 0.5% lidocaine, the skin was cut along the incision line, and the superficial fascial surface of the retroauricular mastoid region was sharply separated. After sufficient hemostasis, a 50-ml skin dilator was placed in the superficial fascial surface detachment area, and a drainage tube was placed. The lower end of the skin dilator was flush with the earlobe of the malformed ear. The incision was sutured (Fig. 1).

**Figure 2:**
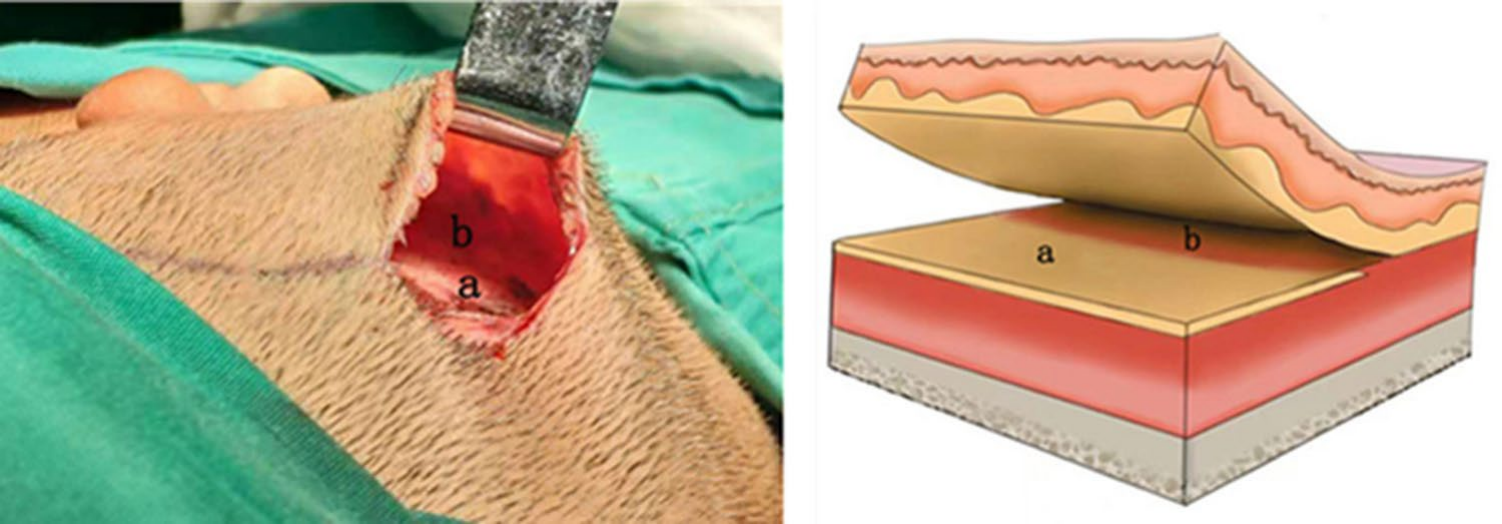
Schematic diagram of the dissection layers in the biplanar surgical method. a: The superficial fascial surface. b: The deep surface of the superficial fascia.

In the experimental group, patients were treated with a skin dilator by the biplanar method. The incision was located 0.5–1 cm behind the patient’s hairline and was approximately 3–5 cm long. The surgical area was designed according to the bare skin area without hair growth in the mastoid region. After local anesthesia with 0.5% lidocaine, the skin was cut along the incision line, and the upper two-thirds of the mastoid region were sharply dissected on the superficial fascial surface. The lower quarter of the mastoid region was sharply dissected from the superficial fascial surface to the deep surface of the superficial fascia. In this way, two planes were formed subcutaneously in the mastoid area. After sufficient hemostasis, the lower third of the 50-ml skin dilator was placed into the deep surface of the superficial fascia, the upper two-thirds of the skin dilator was placed into the superficial fascial surface, and a drainage tube was placed. The lower end of the skin dilator was flush with the earlobe of the malformed ear. The incision was sutured (Fig. 2).

Patients in the experimental and control groups were injected with sterile saline into the dilator on day 7 after dilator implantation. Sterile saline (5–8 ml) was injected every 3 days. The dilators were injected with approximately 70 ml of sterile saline and left to rest for 30 days, and then the second stage of the operation was performed.

### Main outcome measures

The down-moving distance of the skin dilator was measured (using the earlobe of the malformed ear as a reference) and compared between the experimental group and the control group. Complications were monitored, including skin dilator exposure, incision infection, and skin dilator hematoma. The satisfaction of patients and their families was also measured.

### Statistical methods

The SPSSAU data scientific analysis platform (https://spssau.com/) was used for data analysis. Count data are expressed as percentage and number of cases, and the chi-square test was used for comparison between groups. Measurements data are expressed as mean ± standard deviation. If continuous variables conformed to a normal distribution, a t-test was used for comparisons between groups. If data were non-normally distributed, non-parametric tests were used. A *P* value of < 0.05 was considered statistically significant.

## Results

**Figure 3:**
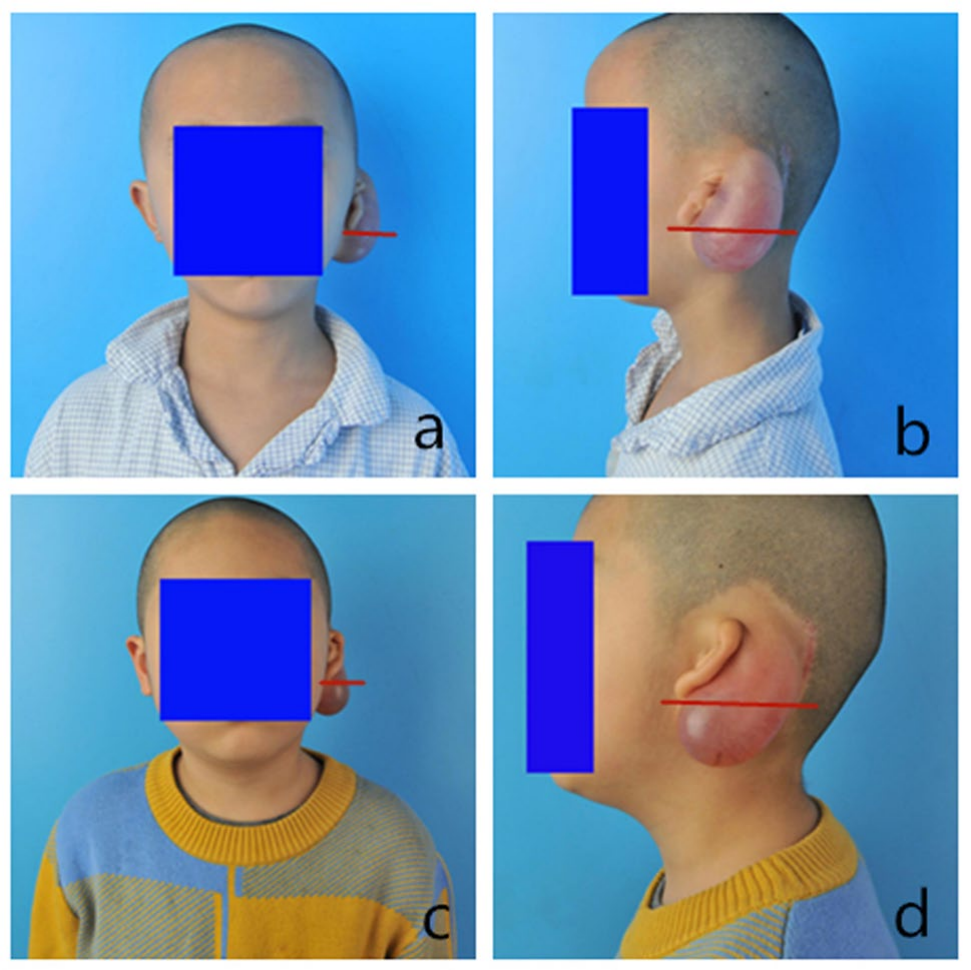
Typical patients in the control group. (**a**) Front view of typical patient 1 in the control group. (**b**) Lateral view of typical patient 1 in the control group. (**c**) Front view of typical patient 2 in the control group. (**d**) Lateral view of typical patient 2 in the control group. The red reference line is the horizontal line of the lowest point of the earlobe of the malformed ear.

**Figure 4:**
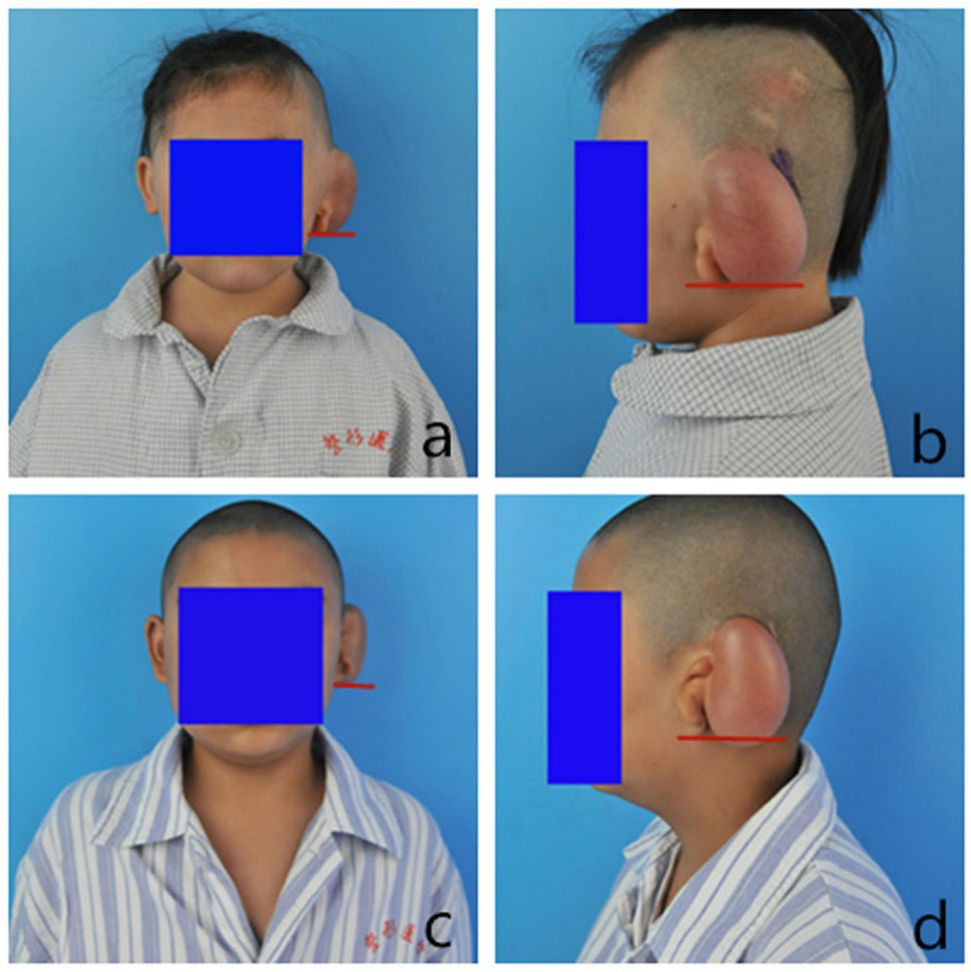
Typical patients in the experimental group. (**a**) Front view of typical patient 1 in the experimental group. (**b**) Lateral view of typical patient 1 in the experimental group. (**c**) Front view of typical patient 2 in the experimental group. (**d**) Lateral view of typical patient 2 in the experimental group. The red reference line is the horizontal line of the lowest point of the earlobe of the malformed ear.

The down-moving distance of the skin dilator was 1.52 ± 0.44 cm in the experimental group and 2.08 ± 0.34 cm in the control group. We used the Kolmogorov–Smirnov test to test the normality of the data and found that the data did not conform to a normal distribution (*P* < 0.05). Thus, non-parametric tests were used to compare the down-moving distance of the skin dilator between the experimental group and the control group. A statistically significant difference was observed in the down-moving distance of the skin dilator between the experimental group and the control group (*P* < 0.05). The down-moving distance of the skin dilator in the experimental group was shorter than that in the control group (Figs. 3 and 4).

In terms of postoperative complications, in the experimental group, 3 patients demonstrated postoperative dilator exposure, 4 patients demonstrated postoperative hematoma, and no patients demonstrated incision infection. In the control group, 4 patients demonstrated postoperative dilator exposure, 3 patients demonstrated postoperative hematoma, and no patients demonstrated incision infection. The chi-square test showed no significant difference in postoperative complications between the experimental group and the control group (*P* > 0.05).

In terms of the satisfaction rate of patients and their families, the satisfaction rate in the experimental group was 95.2%, and the satisfaction rate in the control group was 94.6%. The chi-square test showed no significant difference in the satisfaction rate between the experimental group and the control group (*P* > 0.05).

A summary of the data is shown in Table 1.

**Table 1:** Data summary table.

Variable	Experimental group (n = 63)	Control group (n = 74)	*P* value
Age, years	10.08 ± 2.64	10.16 ± 2.52	*P* > 0.05
**Sex**			
Male	38	43	*P* > 0.05
Female	25	31	
**The site of microtia**			
Left side	41	38	*P* > 0.05
Right side	22	36	
Follow-up time, days	61.81 ± 5.55	62.01 ± 6.09	*P* > 0.05
Down-moving distance of skin dilator, cm	1.52 ± 0.44	2.08 ± 0.34	*P* < 0.05
**Postoperative complications**			
Postoperative dilator exposure	3	4	*P* > 0.05
Incision infection	0	0	–
Postoperative hematoma	4	3	*P* > 0.05
**Satisfaction rate of patients and their families**			
Greatly satisfied	44	42	*P* > 0.05
Satisfied	16	28	
Unsatisfied	3	4	

## Discussion

The mastoid area behind the ear is the main site for skin dilator placement in auricular reconstruction surgery^8^. The skin in this area is tight, the upper and posterior skin is close to the scalp, and the skin is thicker than the lower and anterior skin^9,10^. During expansion of the skin dilator, the pressure of the upper skin on the skin dilator and the gravity of the skin dilator itself cause the position of the skin dilator to move downward. This downward movement of the skin dilator in the retroauricular skin expansion phase results in insufficient expansion of the upper half of the skin dilator and excess expansion of the lower half of the skin dilator. This factor increases the difficulty in embedding the costal cartilage auricle stent in the second stage of auricular reconstruction. Compared with the traditional method of skin expander placement, the biplanar surgical method places the lower one-third of the expander deep into the superficial fascia, increasing the amount of tissue underneath the skin dilator. This method can effectively counteract the pressure of the skin in the mastoid region on the skin expander and gradually increase the gravity of the skin expander itself during the sterile saline injection expansion process, thus reducing the downward movement of the skin expander.

In this study, the skin expander was implanted in the experimental group using the biplanar method, and the skin expander was implanted in the control group using conventional surgery. After analysis, it was found that the down-moving distance of the skin dilator in the experimental group was shorter than that in the control group. There was no significant difference in postoperative complications between the experimental group and the control group. In the comparison of the satisfaction rate of patients and their families, although no significant difference was found with the chi-square test, the satisfaction rate of patients and their families in the experimental group was 95.2%, which is higher than the satisfaction rate of 94.6% observed in the control group. In the process of following up patients, we found that the main factor affecting the satisfaction rate of patients and their families was surgical complications after skin dilator implantation. We strictly followed the principle of asepsis during the operation; thus, there were no cases of postoperative incision infection. However, some patients presented with postoperative dilator exposure and postoperative hematoma. The main causes of postoperative dilator exposure are postoperative hematoma and excessive incision tension during sterile saline injection. The main reasons for the occurrence of postoperative hematoma are incomplete hemostasis of the operator during the operation and vigorous activity by the patients after the operation. Therefore, the main measures to prevent postoperative dilator exposure are prevention of postoperative hematoma and injection of an appropriate volume of sterile saline to prevent excessive incision tension. The main measures to prevent the occurrence of postoperative hematoma are to stop bleeding thoroughly during the operation and for patients to avoid vigorous activity after the operation.

A total of 137 patients were enrolled in this study, and credible conclusions were reached through the rigorous scientific study design and data analysis. However, this study has some limitations that should be noted. First, the experimental group included 63 patients, while the control group included 74 patients. The sample size was small, which affected the credibility of the final conclusions. Second, the researchers were not blinded in this study, which may have impacted the data collection of evaluation indicators. Third, the procedures were not always performed by the same surgeon, and differences in surgical technique may have impacted the surgical efficacy.

In summary, this study found that the treatment effect of biplane skin dilator implantation was better than that of conventional skin dilator implantation. Therefore, biplane skin dilator implantation is worthy of popularization and application in clinical work.

## Supplementary Information

Supplementary Information.

## Data Availability

The data that support the findings of this study are available in the Supplementary Information of this article.
